# Feasibility and Acceptability of Community-Based Telehealth to Prevent Long-Term Care Readmission

**DOI:** 10.1089/tmr.2022.0040

**Published:** 2023-06-26

**Authors:** Jennifer Mallow, Stephen M. Davis, Johnathan Herczyk, Margaret Jaynes, Ben Klos, Marcus Canaday, Laurie Theeke

**Affiliations:** ^1^ School of Nursing, West Virginia University, Morgantown, West Virginia, USA.; ^2^ School of Public Health, West Virginia University, Morgantown, West Virginia, USA.; ^3^ Office of Health Affairs, West Virginia University, Morgantown, West Virginia, USA.; ^4^ School of Medicine, West Virginia University, Morgantown, West Virginia, USA.; ^5^ Take Me Home, West Virginia Bureau for Medical Services, Charleston, West Virginia, USA.; ^6^ School of Nursing, George Washington University, Ashburn, Virginia, USA.

**Keywords:** chronic conditions, home and community-based services, long-term care, readmissions, telehealth

## Abstract

**Background:**

Transitioning to community living after long-term care requires multiple complex individualized interventions to prevent readmission. The current focus of home and community-based services (HCBS) is on increasing consumer engagement and individualizing care. Telehealth interventions provide additional services without the burden of face-to-face encounters and have yet to be evaluated for feasibility and acceptability in rural HCBS.

**Methods:**

West Virginia Bureau for Medical Services and West Virginia University implemented and evaluated a telehealth intervention with 26 Aged and Disabled Waiver or Traumatic Brain Injury Waiver participants who were transitioning back into their communities from a long-term care facility. Feasibility was assessed through recruitment process, fidelity to planned intervention, number of people eligible for participation, number of individuals enrolling in the intervention, enrollment process, completed enrollment, engagement in the intervention, number of weeks participating in the intervention, type of devices provided, attrition, and fidelity to original intervention. Satisfaction with services was used as a marker of acceptability for both participants and providers.

**Results:**

Half (*n* = 12) of the enrolled population completed the full 24-week telehealth monitoring period and modification of the original intervention was necessary for most. Provider and participant satisfaction was high. Recruitment and enrollment may have been affected by COVID-19.

**Conclusion:**

Future implementation will continue to track recruitment and retention efforts. Individualized care plans, demonstration and practice with equipment, family or direct-care worker presence, and live technical support through the phone are needed. Primary care provider and in-home direct-care worker satisfaction workflow planning and evaluation are required.

## Introduction

Transitioning from the inpatient long-term care setting to community living requires in-depth planning and multiple individualized interventions, including (1) precise education related to chronic conditions and ways to seek appropriate levels of care when needed; (2) care coordination to address complex health needs; (3) strategies for building enhanced communication of needs among the individual, caregiver, and care team; (4) a strong collaborative interprofessional team that understands the individualized unique care needs and desires for care; and (5) ongoing assessment, planning, and evaluation of individualized care.^1^

However, a recent publication describing the continuing challenges in rural health in the United States emphasizes that individuals who live in rural areas and suffer disparate conditions such as cancer and mental health issues continue to demonstrate poorer health outcomes due to longer distance to care, lack of timely and appropriate level of care, limited access to specialists, and diminished communication between multiple dispersed caregivers.^2^ These challenges indicate that additional unique interventions are needed to prevent acute and chronic exacerbations of illness and assist those with illness so that they can remain in their home communities.

The Centers for Medicare and Medicaid Services (CMS) instituted the federal Money Follows the Person (MFP) Rebalancing Demonstration with the goal of increasing the use of home and community-based services (HCBS) and reduce the use of institutionally based services (Medicaid.gov).^3^ The long-term goals of this program demonstration include increasing the number of individuals that have transitioned into the community, increasing the length of community residence, decreasing readmission into long-term care, reducing Medicaid and Medicare expenditures, improving quality of care, and increasing quality of life for recipients. Findings of the MFP program today indicate that particular emphasis should be placed on consumer engagement and personalization of supports to achieving these outcomes.^4^

Telehealth interventions offer an effective method for delivering education, assessment, communication, goal setting, and linking dispersed health care teams, especially in rural areas in the time of COVID-19.^5,6^ The safety and quality of telehealth interventions for the treatment of chronic conditions is well established by a strong evidence base. A recent meta-review of 53 systematic reviews examining telehealth interventions for the self-management of long-term conditions such as diabetes, heart failure, asthma, chronic obstructive pulmonary disease, and cancer confirmed the safety of telehealth for managing these conditions, especially type 2 diabetes and heart failure.

No adverse events were noted in any of the included reviews.^7^ Another recent systematic review with meta-analysis further demonstrated the positive impact of nursing led telehealth interventions on the quality of life and self-care ability of patients with chronic diseases.^8^ Despite the demonstrated safety and quality of telehealth for the treatment of chronic conditions, all of the previous telehealth studies included in these reviews occurred in large urban centers such as New York City, Philadelphia, San Francisco, and Ann Arbor (Lee, Lewinsky).^8,9^ This observation is consistent with other research demonstrating that telehealth is underutilized in rural Medicaid populations.^10^

Furthermore, until now, most telehealth interventions aiming to manage acute and chronic conditions, prevent hospital readmissions, and provide mental health services were delivered and administered by health care organizations such as large academic medical centers, the Veterans Administration, primary care provider (PCP) offices, or home health care companies. Thus, the use of telehealth in the community after discharge from long-term care has yet to be evaluated for feasibility, acceptability, and effectiveness. The purpose of this article is to assess the feasibility and acceptability of a home and community-based telehealth program to prevent long-term care readmission.

## Materials and Methods

### Overview

This article reports the feasibility and acceptability of implementing a telehealth pilot (THP) in West Virginia's Take Me Home Transition Program, a federally funded MFP Rebalancing and Demonstration program funded by CMS.

The goal of the Take Me Home Transition Program is to give older adults and people with disabilities greater choice in where to live and receive long-term services and supports. To help achieve the MFP goal, the West Virginia Department of Health and Human Resources, Bureau for Medical Services (BMS) contracted with West Virginia University (WVU) to lead a design team comprising stakeholders and BMS representatives to construct, propose, implement, and evaluate a pilot telehealth intervention. The design and implementation process of this project consisted of three phases. Phase 1, design of the intervention, has been previously published.^1^

In brief, during the original development of this intervention, community stakeholders, health care agencies, state representatives, telehealth experts, and patient advocates followed a process for developing complex interventions and identified two waiver programs, Aged and Disabled Waiver (ADW) or Traumatic Brain Injury Waiver (TBIW), within the state of West Virginia to participate in the THP program. The ADW program is for adults who need nursing home level of care and choose to take part in the ADW program instead of entering a nursing home.

Individuals in the ADW program have at least five areas of need in activities of daily living such as eating, bathing, dressing, grooming, mobility, toileting, and others. The TBIW program is for individuals who score at a Level VII or below on the Rancho Los Amigos Levels of Cognitive Functioning Scale and choose to take part in the TBIW program instead of care at a nursing facility. More information about these programs can be found at the following link: https://dhhr.wv.gov/bms/programs/pages/default.aspx

**FIG. 1.**
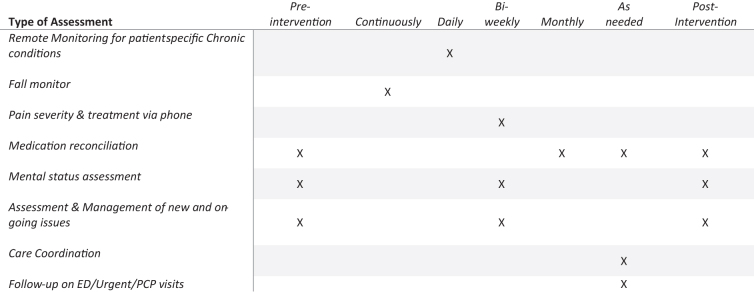
Overview of telehealth intervention. ED, emergency department; PCP, primary care provider.

During Phase 2 of the project, the designed telehealth intervention enrolled 26 adult (aged ≥18 years) ADW or TBIW participants who were transitioning back into their communities from long-term care through the Take Me Home Transition Program. WVU also contracted with two telehealth vendors to provide telehealth equipment and services (remote patient monitoring [RPM] devices and fall monitors) for THP participants and began rolling enrollment and implementation of the THP with interested participants (Fig. 1).

Addressing feasibility, the extent to which the intervention can be implemented as conceptualized, and is plausible in the intended population with desired effects is the purpose of this type of research.^11^ Common measures of feasibility and acceptability were used.^11^ Feasibility was assessed through recruitment process, fidelity to planned intervention, number of people eligible for participation, number of individuals enrolling in the intervention, enrollment process, completed enrollment, engagement in the intervention, number of weeks participating in the intervention, type of devices provided, attrition, and fidelity to original intervention. Satisfaction with services was used as a marker of acceptability for both participants and providers.

### Recruitment, inclusion, and exclusion

ADW and TBIW members accessing Take Me Home Transition Program services were recruited through collaborative efforts by WVU employees and BMS personnel. Participants of the pilot were required to meet the following inclusion criteria to be eligible for enrollment, including 18 years or older, currently receiving services through either the ADW or TBIW Medicaid programs, transitioned through the Take Me Home Transition Program, ability to read and write English, and the ability to, or have someone willing to help with, taking vital sign measurements daily. Exclusion occurred for individuals who were <18 years, not receiving services through either the ADW or TBIW Medicaid programs, transitioned through services other than the Take Me Home Transition Program, were unable or unwilling to take vital sign measurements using provided equipment.

### Assessing feasibility

The process of evaluating feasibility was multifaceted. The study team used field notes to track any issues with recruitment process, enrollment process, and engagement in the intervention. Audits of the planned intervention were conducted to assess fidelity. In addition, the number of people eligible for participation, number enrolling in the intervention, number completing enrollment, and number of weeks participating in the intervention, and number that did not complete the program (attrition) were documented. Finally, the team noted the type of RPM devices provided and fidelity to original intervention.

### Assessing acceptability

Acceptability was measured using the Service User Technology Acceptability Questionnaire (SUTAQ) during the final biweekly assessment call with a Registered Nurse (RN). The SUTAQ assesses the extent to which caregivers or care recipients considered the care to be appropriate based on anticipated and experienced interactions with the monitoring services. The SUTAQ is a psychometrically validated tool that assesses participants' perceptions on benefits (α = 0.89), privacy and discomfort (α = 0.70), care personnel concerns (α = 0.63), satisfaction (α = 0.76), and that technology provided a substitution for in-person care (α = 0.64).^12^ Each of these constructs is a subscale and are scored using a 6-point Likert scale (*1 = Strongly Disagree*, *2 = Moderately Disagree*, *3 = Mildly Disagree*, *4 = Mildly Agree*, *5 = Moderately Agree*, and *6 = Strongly Agree*) with total subscale scores being the median response score.

On the SUTAQ, higher scores are indicative of more agreement with the concept assessed. However, questions on the SUTAQ regarding privacy and discomfort as well as care personnel concerns are negatively worded (i.e., “The kit has made me feel uncomfortable”). Therefore, lower scores on these two subscales indicate disagreement with the concept. In addition, perceptions of enhanced care and increased accessibility of care are categorized together and reported as perceived benefits.

**FIG. 2.**
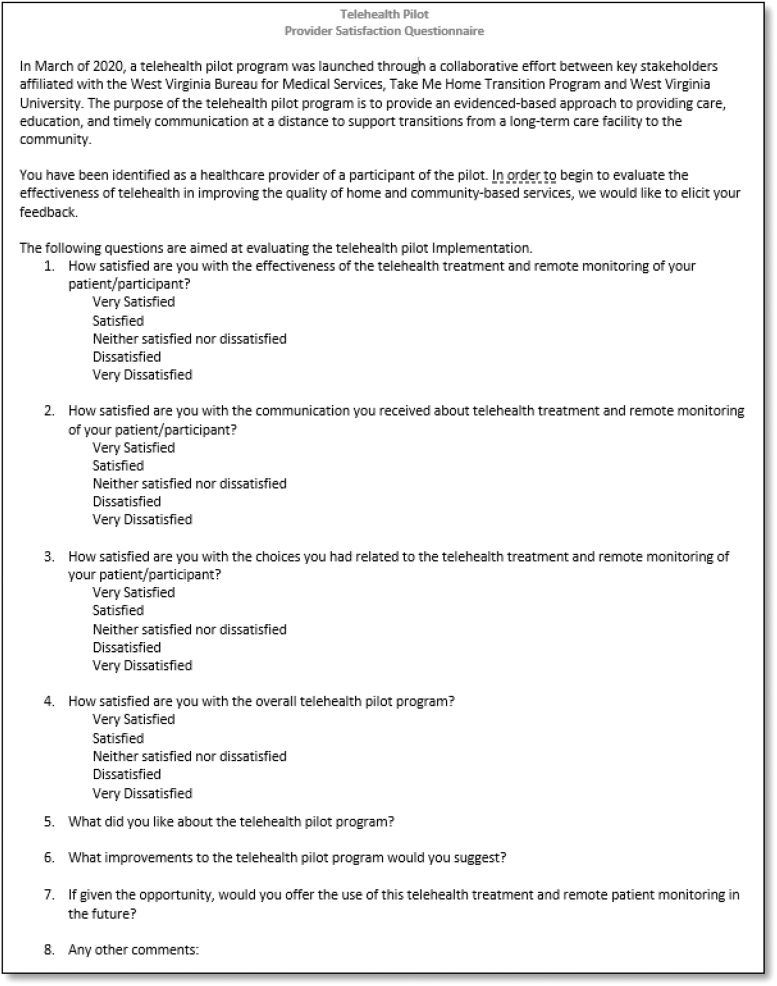
Provider satisfaction questionnaire.

Acceptability and feedback from THP participants' PCP and direct care workers was assessed using a brief 8-item questionnaire (Fig. 2). This questionnaire was created by the evaluation team specifically for this scenario and thus has no measures of validity or reliability. The survey was administered through phone after a participant completed their monitoring period. Four items on this questionnaire assessed satisfaction with effectiveness, communication, provider choice, and the overall program, and were scored using a 5-item Likert scale (*1 = Very Dissatisfied*, *2 = Dissatisfied*, *3 = Neither Satisfied nor Dissatisfied*, *4 = Satisfied*, and *5 = Very Satisfied*). PCPs and direct care workers were also asked four open-ended questions: what they liked, what improvements they suggest, if they would use the program in the future, and any other comments.

### Ethical consideration for human subjects

The West Virginia University Institutional Review Board reviewed and acknowledged the pilot project in January 2020. Additional steps were taken to promote the autonomy of participants by requiring a formal consent process culminating in written consent and Health Insurance Portability and Accountability Act authorization for each participant or their legally authorized representative.

## Results

### Feasibility

The 15-month enrollment period began in March 2020 and ended in May 2021. After being identified as meeting eligibility for either TBIW or ADW services, participants were identified by Transition Coordinators as suitable candidates for telehealth services. Not all candidates were offered telehealth services as originally planned and the clinical judgment of those familiar with the potential participants was trusted regarding the recruitment of participants.

**FIG. 3.**
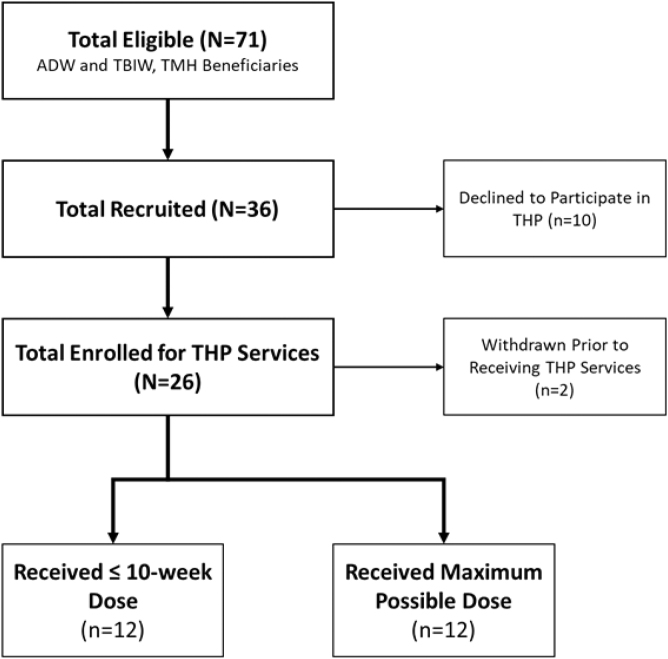
Recruitment and enrollment. ADW, Aged and Disabled Waiver; TBIW, Traumatic Brain Injury Waiver; THP, telehealth pilot; TMH, take me home.

Owing to the COVID-19 pandemic, access to long-term care facilities was limited and likely impacted recruitment. Potential participants were offered the telehealth intervention while currently residing in a long-term care facility. Figure 3 summarizes the recruitment and enrollment efforts. The target was 30 participants. Overall, 36 participants were offered the intervention and 26 participants enrolled into the pilot. Only 71 Take Me Home Transition Program beneficiaries transitioned during the recruitment period. Therefore, recruitment efforts reached approximately half of those eligible. Although recruitment occurred from both ADW and TBIW populations, only one TBIW participant was enrolled, with the rest being ADW. Hence, comparisons between groups were not possible.

Once enrolled, the most recent nursing assessment of the individual was obtained from the long-term care facility and used to develop an individual care plan for RPM devices and education. In addition to a base unit, which transmitted the monitoring data and facilitated educational modules, all THP participants received a blood pressure cuff to assist with home monitoring. Most participants (92%, *n* = 24) received a pulse oximeter (PulseOx). Six THP participants received equipment to monitor glucose levels; two participants were provided a scale to monitor weight. Thermometer readings were self-reported using non-Wi-Fi enabled devices provided to two participants.

Monitoring equipment was directly mailed to participants with a goal of starting services within 2 days of transitioning home. Only five participants were able to meet the 2-day time frame. In response, this benchmark was modified. Although equipment was still provided to participants within 2 days of transitioning home, a period of 2 weeks was set as a goal to have the monitoring equipment active. Out of the remaining 21 participants, only one did not meet this goal. After several attempts to assist and motivate the participant who did not meet this goal for use of both the RPM and telephone services, the participant was withdrawn from the pilot. Out of the 26 enrolled participants, 5 needed and received assistance from a family member or in-home direct care worker to set up monitoring equipment.

Participant data from the remote devices were sent to RNs employed by the telehealth equipment vendor and abnormal findings were escalated as needed to the RN of the pilot for coordination of care. This care included the provision of education, revision of care plans, or consultation with the participant's PCP. Escalations of care that could not be treated in this way resulted in coordination with family members, sending participants to urgent care, or by calling 911 when clinically indicated. Home monitoring continued in this manner from the first transmission of RPM for up to 6 months. The equipment was returned to the vendor through FedEx delivery. Post-telehealth intervention evaluation was conducted related to loneliness and telehealth satisfaction.

### Retention

**FIG. 4.**
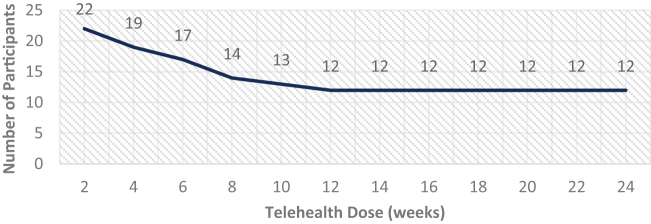
Total telehealth population retention.

**FIG. 5.**
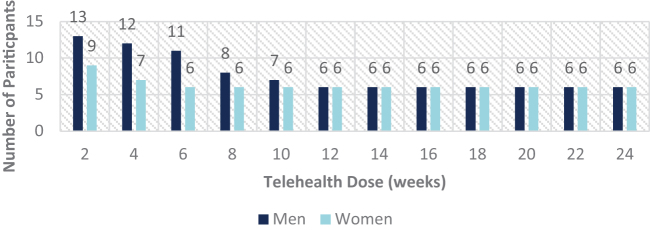
Gender-specific telehealth population retention.

The total telehealth group retention rate is depicted in Figure 4. Overall, half (*n* = 12) of the enrolled population completed the full 24-week telehealth monitoring period, and 100% of participants that received a 12-week dose of telehealth completed the entire 24-week pilot. Nearly 92% (*n* = 22) received at least 2 weeks of telehealth monitoring before a slow, but gradual, attrition rate was noticed. More men (*n* = 7) withdrew compared with women (*n* = 3), as depicted in Figure 5. On average, men who withdrew were younger (60.7 years) compared with their counterparts who completed the full 24-week dose (mean age of 62.3 years).

The opposite age finding was noted in women who withdrew (58.0 years) when compared with those who received a full 24-week dose (53.2 years). Reasons for attrition from the THP fell into three main themes: loss of eligibility for TBIW and ADW services, lack of adherence to RPM and/or audio only assessments, and loss of interest. One participant shared that she no longer wished to participate because she felt that it was “…too difficult and time intensive.” After 10 weeks, another participant stated they “…were not able to setup monitoring and do not believe it is of any use.”

### Acceptability

Participants were asked to monitor daily; however, some participants only monitored a few times per week. Seven participants utilized the telehealth equipment and monitored vitals daily or close to daily. Nearly 33% (*n* = 8) of participants monitored two to three times per week. In response to low adherence to monitoring, participants were asked to use the telehealth monitoring equipment at least every 3 days; if the participant did not transmit vital measures after 3 days, they would receive a call to assess the reasons for nonadherence. Participants who were called regarding lack of transmissions stated that several factors contributed, including busy lifestyle, doctor visits, lack of physical assistance, and equipment failure as reasons for monitoring less often than requested. Heart rate, blood pressure, and oxygen saturation readings were the most frequently transmitted followed by glucose, weight, and temperature measurements (Table 1).

**Table 1.** Biometric Transmission Frequencies

*Biometric reading*	*Number of transmissions*
Pulse	1609
Temperature	7
PulseOx	1083
Weight	66
Glucose	345
Blood pressure	1100
Total	4219

In addition, almost half (46%, *n* = 11) of participants were provided with a fall monitor and emergency alert services. Outside of initial and accidental activations, no emergency alerts were sent using the provided devices. Hence, it is unclear if any of the fall monitor devices continued to be used on a daily basis.

### SUTAQ responses

**FIG. 6.**
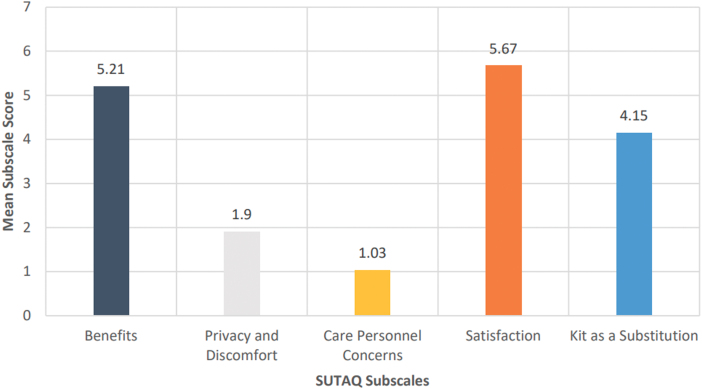
Mean SUTAQ scores (*n* = 13).

Mean scores for participants (*n* = 13) who provided responses to the SUTAQ are shown in Figure 6. Participants completed the SUTAQ at the end of their participation in the trial. In addition, aggregate descriptive statistics for each of the 22 items on the SUTAQ are included in Table 2. From the aggregate mean subscale scores hereunder, participants moderately agreed that the equipment and services provided were beneficial and reported being satisfied with the services. Participants also moderately disagreed that the equipment was intrusive or that they had concerns regarding privacy and confidentiality of their health information. Likewise, there were low concerns about the qualifications of and continuity of care provided by the health professionals providing monitoring and coordination services.

**Table 2.** Mean Service User Technology Acceptability Questionnaire Item Scores

*Subscale*	*Item*	****N****	*Range*	*Mean*	*SD*	*Median*	*IQR*
Perceived benefits	The kit I received has increased my access to care (health and/or social care professionals)	13	2–6	4.7	1.1	5	1
	The kit I received has helped me to improve my health	13	3–6	5.3	1	6	1.5
	The kit I received has saved me time that I did not have to visit my general practitioner's clinic	13	3–6	5.3	1	6	1.5
	The kit has made it easier to get in touch with health and social care professionals	13	3–6	5.1	0.9	5	1.5
	The kit has made me more actively involved in my health	13	2–6	5	1.2	5	2
	The kit allows the people looking after me, to better monitor me and my condition	13	2–6	5.1	1.2	6	1.5
	The kit can be/should be recommended to people in a similar condition to mine	13	1–6	4.8	1.6	6	2
	The kit can certainly be a good addition to my regular health or social care	13	2–6	5.4	1.2	6	0.5
	The kit has allowed me to be less concerned about my health and/or social care	13	4–6	5.7	0.6	6	0
Privacy and discomfort	The kit I received has interfered with my everyday routine	13	1–6	2.3	1.2	2	1
	The kit I received has invaded my privacy	13	1–3	1.5	0.6	1	1
	The kit has made me feel uncomfortable, e.g., physically or emotionally	13	1–5	2.1	1.3	2	1
	The kit makes me worried about the confidentiality of the private information being exchanged through it	13	1–4	1.5	0.8	1	1
Care personnel concerns	I am concerned about the level of expertise of the individuals who monitor my status through the kit	13	1–6	2.6	2.1	2	4
	The kit interferes with the continuity of the care I receive (i.e., I do not see the same care professional each time)	13	1–4	1.7	1	1	1.5
	I am concerned that the person who monitors my status, through the kit, does not know my personal health/social care history	13	1–4	1.3	0.8	1	0.5
Perceived satisfaction	The kit has been explained to me sufficiently	13	4–6	5.6	0.6	6	0.5
	The kit can be trusted to work appropriately	13	3–6	5.1	0.9	5	1.5
	I am satisfied with the kit I received	13	2–6	5.3	1.1	6	1
Kit as a substitution	The kit can be a replacement for my regular health or social care	13	2–6	3.8	0.9	4	1
	The kit is not as suitable as regular face-to-face consultations with the people looking after me	13	2–5	3.4	1.1	4	2
	The kit has allowed me to be less concerned about my health status	13	1–6	5.1	1.4	6	1.5

IQR, interquartile range; SD, standard deviation.

#### Perceived benefits

Participant perceptions of benefits from the equipment used in the pilot were assessed using a nine-item subscale on the SUTAQ. Questions about perceived access to care, self-rated improvements in health, and ease of communication with their health care providers are included on this subscale. Overall, participants moderately agreed that the telehealth equipment was beneficial with an average score on this subscale of 5.2 (standard deviation [SD] = 1.0).

#### Privacy and discomfort and care personnel concerns

Privacy and discomfort with the monitoring technology were assessed with a four-item subscale of the SUTAQ. A low average score of 1.9 (SD = 0.94) indicates moderate participant *disagreement* that the equipment caused overt discomfort. Both men ($\bar{x}$ = 1.2, SD = 0.5) and women ($\bar{x}$ = 1.6, SD = 1.0) indicated strong disagreement that they had concerns about the confidentiality of their information being shared through the monitoring equipment. Participants also indicated disagreement that the monitoring equipment invaded their privacy. These observations indicate that confidentiality and privacy concerns, a major influence of telehealth adoption in other populations,^13^ are not a concern among the ADW and TBIW recipients, thus supporting telehealth adoption.

In addition, concerns about the continuity of care and the skills of those providing and coordinating that care were assessed on a 3-item subscale. Overall, the participants had minimal concern about the expertise of professionals providing telehealth monitoring with an average score on the Care Personnel Concerns SUTAQ subscale of 1.92 (SD = 1.0).

#### Perceived satisfaction

Participants indicated strong satisfaction with the telehealth equipment, with a high average score of 5.42 (SD = 0.92) on the 3-item SUTAQ subscale. Both men (5.8, SD = 0.41) and women (5.6, SD = 0.55) strongly felt that the technology had been explained clearly and sufficiently to them. Men moderately agreed that the equipment could be trusted to work properly (5.5, SD = 0.84), whereas women moderately agreed (4.8, SD = 1.6) that the equipment functioned appropriately.

#### Kit (technology) as a substitution

The average score of 4.21 (SD = 0.52) indicates that participants mildly agreed that the technology could be used as a replacement for their usual care received. A finding to note is that participants moderately agreed (5.1, SD = 1.3) that the monitoring services and equipment provided allowed them to be less concerned about their health status. Despite this perceived benefit from the telehealth equipment provider, this 3-item subscale identified that participants did not feel the monitoring technology would be suitable as a replacement to face-to-face consultations with care providers (3.5, SD = 1.3).

#### Provider acceptability of the pilot

Four PCPs responded to phone calls and provided responses related to their satisfaction and experience with the THP. However, when a PCP call was needed related to participant care, most were answered by the PCP's staff member and the message transferred to PCP. The PCPs were satisfied with the effect of the pilot on their patient (4.5, SD = 0.5) as well as the choices for care that resulted from participation in the THP (4.5, SD = 0.5). These scores indicate that given the opportunity, they would offer the use of telehealth and RPM in the future. When asked to provide suggestions for improvements to the THP, the prevailing theme was additional assistance with the initial setup of devices. All respondents indicated they would recommend telehealth services for future use.

Four direct care workers responded to phone calls and shared their experiences. Similar to the PCPs, results available from the direct care workers are favorable with an average satisfaction of the effects from the telehealth intervention of 4 (SD = 1.4), an average satisfaction with communication of 5 (SD = 0), choices provided by the THP of 4 (SD = 1.4), and an average overall satisfaction with the THP of 4.6 (SD = 0.4).

One direct care worker did express dissatisfaction with the effect and choices for care resulting from the THP. When provided the chance to elaborate, the health worker explained that they were unaware of the member's participation in the pilot and believed that the RN was a patient advocate. The direct care worker was unaware of any equipment provided to the THP participant and, therefore, was unable to assist with monitoring. Although uninformed about the THP, the direct care worker was very satisfied with the nurse, who was available to assist with care coordination.

## Discussion

This is one of the first pilot programs to use telehealth services in the HCBS waiver population. Despite the onset of the COVID-19 pandemic, to the pilot recruited 87% of the planned target sample (26 of 30) with complete data available for 93% of the enrolled. Results indicated high patient and provider satisfaction, although modifications to the planned telehealth protocol, especially the frequency of RPM, were needed to match patient preferences. Length of participation was variable before 12 weeks.

One key finding is that patients participating for at least half of the planned pilot length (i.e., 12 weeks) participated for the entire 6 months, which may suggest the presence of patient and environmental characteristics that predict full adoption of telehealth. This finding is particularly important as patients and health care organizations were required, due to the COVID-19 pandemic, to pivot to telehealth. It is important to advance the understanding of factors that influence full adoption of telehealth technologies so that they can be effective for people with multiple chronic conditions and limited health care access.

Our finding that more men withdrew early from the pilot is consistent with a study of Alabama Medicaid claims during the CoVID-19 pandemic where women were more likely to have telehealth visits after the onset of the pandemic, despite having a similar rate of visits before the pandemic.^14^ However, our study specifically focused on Medicaid waiver recipients transitioning home from an institution versus the entire Medicaid population. A very recent systematic review also revealed that being female was more likely to be facilitator to the adoption of m-health. This same review also indicated lack of technical skills, being single or living alone, having lower income, living in rural areas, being older, and having a lower level of education as prominent barriers to the adoption of m-health,^15^ which may explain the fact that only half of our participants completed the entire 6-month dose.

In addition, another recent review on the adoption of telehealth technologies emphasized the need for preparation on the part of health care systems to facilitate full adoption. These needs include addressing knowledge gaps, capacity building in the workforce, funding, and identifying ways to handle restricted information.^16^ As researchers work to make care more precise and improve health inequity by improving access, international efforts are being made to improve use of telehealth by advising through policy recommendations to minimize regulatory restrictions so that people can get the care they need where they need it.^17^

Recruitment efforts may have been impacted due to the COVID-19 pandemic as recent literature indicates that post-acute care discharges to skilled nursing facilities decreased during the pandemic.^18^ Limited access to long-term care facilities due to the pandemic also likely impacted recruitment of participants. In addition, the acute and overwhelming need to respond to the pandemic in long-term care in the region of this study likely diminished the overall total number of potentially eligible participants who were able to transition home.^19^

During the pandemic, long-term care facilities were not discharging patients and remained at capacity or over capacity in some cases, rendering them unable to admit patients as well, further diminishing the number of potential participants. Decisions related to when and how to be transitioned home during the pandemic may have also been altered and limitations on visitation may have prompted families to transition individuals more quickly than anticipated. Conversely, fear of transmission of illness to elderly or those with chronic conditions may have prompted prolonged transition to the community.

Frequent evaluation of the effectiveness and acceptability of individual care plans is necessary. These individual care plans were developed based on ideal standards of care. However, participants found adhering to daily self-monitoring onerous. Qualitative findings from a previous study suggest that daily home monitoring may be a “threat to identity and independence.”^20^ These findings suggest that monitoring daily places emphasis on the participant's chronic condition and not on living a full and rewarding life in the community. Adjustments in the care plan were made in conjunction with the participant that accounted the freedom of individual lifestyle. Monitoring once or twice a week was more desirable to some participants and some desired no RPM but found phone assessment by a nurse acceptable. Thus, balancing dose of telehealth while respecting patient autonomy is required.

Facilitating the adoption of telehealth will require that equipment to be used after discharge is demonstrated with verbal and written instructions, along with practice sessions before discharge. In any population, transition from a long-term care setting to a more independent living situation in the home community can be overwhelming and present unanticipated challenges. Adding new technological devices for remote monitoring during this transition may add burden instead of the intended support. Planning the right time to introduce the equipment and facilitate learning the equipment will be critical to adoption of the telehealth modality.

Receiving care support that is precise to an individual's needs, provided at an appropriate time, and provided at the location of their choosing could promote a sense of control, sense of coherence, empowerment, and improve quality of life.^21^ It is known that sense of coherence is reflective of a person's ability to deal with the stressors of everyday life. If we work to enhance the comprehensibility, manageability, and meaningfulness of telehealth efforts, it is more likely that people will be motivated to adopt the technology as a tool for promoting health and longevity.

Planning for equipment failure is also critical to telehealth adoption. Patients need to have access to live technical support for setting up devices through phone or in person based on these findings. Troubleshooting technology failures is not intuitive to many people and for those who suffer multiple chronic conditions or traumatic brain issues, help with troubleshooting is needed. Arranging for someone to be physically present in the home for equipment setup before discharge would be beneficial and may prevent frustration for the participant. These findings are congruent with the findings in this population related to use of technology in the community.^22^

### Limitations

The SUTAQ was completed at the end of each participant's 6-month intervention. Other telehealth trials used the SUTAQ with both technology users and nonusers and found that nonusers reported dissatisfaction and low acceptability.^23^ Only one individual who did not complete the intervention answered the satisfaction questionnaire. Hence, most of the satisfaction ratings are for those who completed the trial and could be positively skewed.

The finding that telehealth is valuable as an addition to face-to-face but not a replacement for face-to-face care for chronic conditions is supported in the literature.^24^ Providers in this pilot program included the participants' PCP and direct care workers. This intervention was different than traditional telehealth programs because the telehealth services were provided outside the health care system as an additional service. Before implementation, it was not known how this service would be perceived by PCPs. In addition, home health care workers were under an enormous additional burden due to the pandemic.^25^ Hence, it was unknown how implementation of this project would impact their workflow and satisfaction. Satisfaction from both groups was positive; however, more work remains before scaling this intervention. A process for contacting PCPs and scheduling follow-up care is recommended. In addition, providing additional information to direct care workers without adding burden to their workflow is necessary.

## Conclusion

Future implementation will continue to track recruitment efforts and develop individualized care plans that consider burden and preferences of participants. Dose of RPM is a consideration and demonstration and practice with equipment before discharge is needed. In addition, having assistance in the home from family or a care worker when equipment is set up and used in addition to live technical support through telephone may improve satisfaction and adherence. An assessment of satisfaction with the equipment after practice should be completed to determine issues and barriers to implementation before transition home. Although PCP and direct care worker satisfaction was high, additional focus on workflow is needed before increasing the number of participants using telehealth to prevent long-term care readmission.

## Abbreviations Used

ADW: Aged and Disabled Waiver
BMS: Bureau for Medical Services
CMS: Centers for Medicare and Medicaid Services
ED: emergency department
HCBS: home and community-based services
IQR: interquartile range
MFP: Money Follows the Person
PCP: primary care provider
RN: Registered Nurse
RPM: remote patient monitoring
SD: standard deviation
SUTAQ: Service User Technology Acceptability Questionnaire
TBIW: Traumatic Brain Injury Waiver
THP: telehealth pilot
WVU: West Virginia University
